# An Analysis of Ophthalmic Plastic and Reconstructive Surgery Fellowship Program Directors in the United States

**DOI:** 10.7759/cureus.26268

**Published:** 2022-06-23

**Authors:** Parth A Patel, Prem N Patel, Harris Ahmed, Parsha Forouzan

**Affiliations:** 1 Ophthalmology, Augusta University Medical College of Georgia, Augusta, USA; 2 Ophthalmology, University of Texas Southwestern Medical Center, Dallas, USA; 3 Ophthalmology, Loma Linda University Medical Center, Loma Linda, USA

**Keywords:** program director, national institutes of health, h-index, leadership, oculoplastic surgery

## Abstract

Purpose

This study aims to examine the demographic features, academic backgrounds, and scholarly achievements of ophthalmic plastic and reconstructive surgery (OPRS) fellowship program directors (PDs) in the United States.

Methods

In this cross-sectional analysis, publicly accessible sources were accessed in March 2022 to collate the demographic and academic profiles of PDs of American Society of Ophthalmic Plastic and Reconstructive Surgery (ASOPRS)-accredited OPRS fellowships. Differences by gender and program rank were assessed using the Mann-Whitney Utest.

Results

Fifty-four PDs were identified, the majority of whom were males (88.89% (n = 48)). The average age was 58.48 years. Of the PDs, 96.3% (n = 53) obtained a medical degree, and all completed residency training in the United States. In addition, 9.26% (n = 5) had another degree, either a Doctor of Philosophy (n = 3) or master’s degree (n = 2). A substantial proportion of individuals completed medical school (20.37% (n = 11)), residency (20.37% (n = 11)), or fellowship (31.48% (n = 17)) at an institution affiliated with the program where they were PDs. The most common additional fellowship obtained was neuro-ophthalmology (16.67% (n = 9)). The average h-index was 19.30 (range, 0-60), average five-year h-index was 4.85 (range, 0-36), and average m-quotient was 0.63 (range, 0-2.22). A significant difference in the median five-year h-index was observed between females and males (7 (range, 3-36) versus 4 (range, 0-10); p= 0.038).

Conclusions

This analysis indicated that OPRS PDs in the United States were principally males with extensive scholarly productivity. As women remain underrepresented in OPRS, increased gender parity at this leadership position should be encouraged in order to expand the recruitment of women into the field.

## Introduction

An increasingly greater proportion of graduating ophthalmology residents are pursuing fellowship opportunities. Between 1987 and 2003, the proportion of residents who sought such training increased from 43% to 67% [1], a figure that has expanded further in the most recent 2022 match [2]. This trend reflects myriad considerations including more substantial prospects upon officially entering the workforce, a specialized skillset, and greater ostensible prestige [1].

Currently, no ophthalmology fellowship in the United States is accredited by the American Council of Graduate Medical Education (ACGME), although the American Society of Ophthalmic Plastic and Reconstructive Surgery (ASOPRS) accredits oculoplastic and reconstructive surgery (OPRS) fellowship programs [3]. As fellowship program directors (PDs) have the immense responsibility of operating and improving their programs, there has been significant interest in assessing the characteristics associated with this leadership position, with analyses conducted of PDs in cornea and glaucoma [4,5]. However, hitherto, no investigations have described the demographic attributes, educational backgrounds, and academic accomplishments of ASOPRS-accredited fellowship PDs in the United States. Our objective was to provide an analysis of these features and examine differences according to gender and fellowship program rank.

## Materials and methods

In this cross-sectional analysis, all ASOPRS-accredited fellowship PDs in the United States were identified from the society’s domestic fellowship directory. This database was accessed in March 2022.

PD characteristics including age, gender, medical school attended, graduation year, residency and fellowship location, degrees obtained, academic rank, and awards received were obtained from publicly available sources such as institutional websites, online curriculum vitae, and databases (e.g., Healthgrades, Doximity, and LinkedIn). The amount of clinical experience for each PD was approximated by calculating the number of years since medical school completion. This methodology has been previously employed in other analyses of leadership in ophthalmology [4-7]. Program rankings were obtained from the US News Best Hospitals for Ophthalmology to designate the “top 10” programs.

Using the Scopus database, each PD’s scholarly activity, including total publications, citation count, h-index, five-year h-index, year of first publication, and m-quotient (calculated as the h-index divided by the number of years since first publication), was collated. By integrating a temporal correction, the m-quotient is useful to limit the influence of experience on scholarly productivity. Lifetime National Institutes of Health (NIH) funding and the number of awarded grants were obtained from the NIH Research Portfolio Online Reporting Tools Expenditures and Results (RePORTER) module. Editorial (and advisory) board membership was identified for prominent general ophthalmology journals (Ophthalmology, JAMA Ophthalmology, and American Journal of Ophthalmology) and OPRS-specific journals (Ophthalmic Plastic and Reconstructive Surgery and Orbit). All data were collected in March 2022.

As this study utilized publicly available data, institutional review board approval was not required. This investigation adhered to the tenets of the Declaration of Helsinki.

Comparisons between groups were assessed using the Mann-Whitney U test with a threshold for significance set at p < 0.05. All analyses were performed using GraphPad Prism version 9 (San Diego, CA, USA).

## Results

Overall, we identified 54 ASOPRS-accredited OPRS fellowship PDs in the United States. The demographic characteristics, educational backgrounds, and scholarly achievements of PDs are described in Table 1.

**Table 1:** Characteristics of ASOPRS-Accredited OPRS Fellowship Program Directors ASOPRS: American Society of Ophthalmic Plastic and Reconstructive Surgery, OPRS: ophthalmic plastic and reconstructive surgery, SD: standard deviation, PhD: Doctor of Philosophy, USA: United States of America, NIH: National Institutes of Health ^a^Proportion of program directors who completed medical school, residency, fellowship, or all three at an institution affiliated with the program at which they served as fellowship program director

Demographic Characteristics	
Age (Mean ± SD)	58.48 ± 9.55 years
Clinical Experience (Mean ± SD)	32.78 ± 10.13 years
Female (% (n))	11.11% (6)
Educational Backgrounds	
MD (% (n))	100% (54)
PhD (% (n))	5.56% (3)
Master’s Degree (% (n))	3.70% (2)
Medical School in the USA (% (n))	96.30% (52)
Residency in the USA (% (n))	100% (54)
Additional Fellowship (% (n))	29.63% (16)
Length of Subspecialty Training (mean ± SD)	1.83 ± 0.67 years
Program Affiliations^a^	
Medical School (% (n))	20.37% (11)
Residency (% (n))	20.37% (11)
Fellowship (% (n))	31.48% (17)
All (% (n))	9.26% (5)
Academic Positions	
Affiliate/Adjunct or No Academic Rank (% (n))	12.96% (7)
Assistant Professor (% (n))	1.85% (1)
Associate Professor (% (n))	29.62% (15)
Professor (% (n))	53.70% (29)
Department Chair (% (n))	3.70% (2)
Endowed Positions (% (n))	16.67% (9)
Scholarly Achievements	
Publications (Mean ± SD)	92.78 ± 72.14
H-Index (± SD)	19.30 ± 10.72
Five-Year H-Index (± SD)	4.85 ± 5.11
M-Quotient (± SD)	0.63 ± 0.32
NIH Funded (% (n))	7.41% (4)
Average NIH Funding (mean ± SD)	$1,829,632 ± $1,052,847
Heed Fellows (% (n))	12.96% (7)
Editorial Board Members (% (n))	22.64% (12)

Analyses of ASOPRS-accredited OPRS fellowship PDs by gender and program ranking are presented in Table 2.

**Table 2:** Differences Among ASOPRS-Accredited OPRS Fellowship Program Directors by Gender and Program Ranking ASOPRS: American Society of Ophthalmic Plastic and Reconstructive Surgery, OPRS: ophthalmic plastic and reconstructive surgery ^a^p-value assessed using the Mann-Whitney U test ^b^US News Best Hospitals for Ophthalmology was used to identify the top 10 hospitals

Gender Comparisons	Female	Male	P-Value^a^
Age (Median (Range))	58.5 (53-68) years	57.5 (42-77) years	0.57
Clinical Experience (Median (Range))	32 (28-44) years	33 (18-52) years	0.62
Publications (Median (Range))	104 (24-328)	75 (0-377)	0.38
H-Index (Median (Range))	23 (14-60)	18 (0-50)	0.16
Five-Year H-Index (Median (Range))	7 (3-36)	4 (0-10)	0.04
M-Quotient (Median (Range))	0.66 (0.32-2.22)	0.63 (0-1.28)	0.52
Ranking Comparisons^b^	Top 10 Programs	Other Programs	P-Value
Age (Median (Range))	65.5 (40-72) years	56 (43-77) years	0.27
Clinical Experience (Median (Range))	39 (16-46) years	31 (18-52) years	0.33
Publications (Median (Range))	117 (16-377)	72 (0-328)	0.11
H-Index (Median (Range))	22 (9-50)	18 (0-60)	0.14
Five-Year H-Index (Median (Range))	5.5 (1-10)	4 (0-36)	0.18
M-Quotient (Median (Range))	0.64 (0.32-1.28)	0.62 (0-2.22)	0.37

## Discussion

Our analysis of ASOPRS-accredited OPRS PDs in the United States provided insights into their demographics, academic backgrounds, scholarly achievements, and differences by gender and program rank. We observed considerable variation in these characteristics, although generally OPRS fellowship PDs were older males who previously graduated from allopathic schools in the United States, had an academic affiliation as a full professor, and possessed extensive scholarly productivity. These findings should serve as useful benchmarks for furthering our understanding of the current state of ophthalmology leadership and for future comparisons of its evolution.

Males comprised the majority of PDs included in this investigation, similar to other studies of ophthalmology department chairs, residency program directors, and cornea and glaucoma fellowship directors [4-7]. However, compared to the latter three cohorts, the proportion of females among ASOPRS-accredited OPRS fellowship PDs was markedly lower. This difference may partially be attributed to the smaller number of women pursuing ASOPRS-accredited OPRS fellowships. An investigation of Canadian ophthalmologists noted that females were significantly more likely to pursue training in pediatric ophthalmology and strabismus, whereas males were more likely to pursue training in vitreoretinal surgery and OPRS [8]. Although Azad et al. reported improved gender parity in ASOPRS membership since its founding, female representation among leadership positions remained limited [9]. Multiple investigations have demonstrated that same-sex mentors are crucial for the recruitment and retention of female faculty [10,11]. Thus, the pipeline for women entering OPRS may be bolstered by promoting their equity in leadership positions in the field. Interestingly, our study further revealed that female PDs had significantly higher five-year h-indexes compared to their male counterparts, suggesting a trend toward greater research productivity among the former group in more recent years. This is consistent with broader findings from other investigations that have noted a greater number of female authors among OPRS-specific journals and presenters at ASOPRS meetings [12,13].

The educational backgrounds of ASOPRS-accredited OPRS fellowship PDs varied. A notable proportion of individuals completed their medical degree, residency, and/or fellowship at an institution associated with the program they led. A similar degree of continuity has been reported for glaucoma fellowship PDs [4], indicating a relationship between the institutions where leadership in ophthalmology trained and where they eventually became faculty. Additional fellowships were not uncommon, with a notable proportion of individuals trained in neuro-ophthalmology. This is unsurprising considering the degree of overlap that exists with the management of complex patients between both subspecialties, particularly in academic settings. Unexpectedly, only 9% of ASOPRS-accredited OPRS fellowship PDs had an additional advanced degree, which constitutes a smaller proportion of individuals relative to ophthalmology department chairs (28%) [7], residency program directors (20%) [6], and glaucoma fellowship PDs (23%) [4]. It is unknown why this phenomenon was observed. One potential explanation may be that oculoplastic surgeons with PhD degrees do not possess adequate time to devote to administrative responsibilities in addition to their clinical and research duties. However, further research would be required to clarify this trend.

Most ASOPRS-accredited OPRS fellowship PDs held an academic appointment, primarily as full professors (53.7%). Compared to their colleagues in cornea (34.1%) and glaucoma (41%) [4,5], the proportion of full professors was higher, which may suggest differences in academic advancement between subspecialty leadership.

One major factor affecting ascension to leadership positions is academic output [6,7]. Overall, the PDs included in our investigation were exceptionally productive, with an average of 92.78 publications. However, considerable variation in this figure (standard deviation, 72.14) suggests discrepancies in the criteria programs considered when hiring PDs. We additionally included the h-index and m-quotient, which are measures of an individual’s lifetime and yearly scientific achievement, respectively [14]. Collectively, the h-indexes of ASOPRS-accredited OPRS PDs (19.3) were larger than those of ophthalmology residency program directors (8.7) and cornea PDs (16.5) and lower than those of ophthalmology department chairs (24) [5-7]. As no similar studies had previously reported the m-quotient, we were unable to compare this metric across the ophthalmology leadership; however, this value should be useful to evaluate future trends in yearly academic productivity among PDs. Interestingly, Heed Fellows comprised a smaller percentage of ASOPRS-accredited OPRS fellowship PDs (12.96%) compared to cornea (20.5%) and glaucoma PDs (20%) [4,5]. The Heed Fellowship is a prestigious postgraduate award that supports aspiring clinician-scientists pursuing academic careers in ophthalmology [15]. These disparities potentially suggest distinct academic interests for individuals pursuing an OPRS fellowship.

We acknowledge some limitations with our investigation, particularly with its dependency on online resources. Only ASOPRS-accredited fellowship PDs were identified, thereby excluding a subset of non-accredited fellowship PDs. Furthermore, data that were extracted from publicly accessible sources were not verified by contacting each program. However, as most information was acquired from institutional websites, we believe these data to be largely accurate. In addition, the Scopus database may not have captured all publications, thereby leading to an underestimation of each PD’s scholarly productivity. Finally, this study aimed to descriptively assess the characteristics of OPRS fellowship PDs rather than compare them to their non-PD colleagues, thereby precluding conclusions regarding differences between these groups.

## Conclusions

In summary, ASOPRS-accredited OPRS fellowship PDs are predominantly male allopathic graduates with substantial scholarly output. Although an increasing number of women enter the field, female representation among PDs remains limited. Future efforts should concentrate on providing opportunities for the advancement of females in leadership positions, particularly through the expansion of mentorship networks. While a considerable number of PDs were involved in research, there is significant variability among metrics, indicating that scholarly productivity may not be necessary for an appointment at every program. Further analysis of trends in this position and in other academic leadership positions would offer insights to individuals interested in a career in ophthalmic medical education.
